# Etomidate in Severe Cushing Syndrome: A Systematic Review

**DOI:** 10.1210/jendso/bvaf039

**Published:** 2025-03-19

**Authors:** Dimuthu Tharanga Muthukuda, Kamani Dhanushka Liyanaarachchi, Kushalee Poornima Jayawickreme, Pasyodun Koralage Buddhika Mahesh, Vidana Gamage Dinithi Ruwanga, Sinduja Kumar, Chandrika Subasinghe, John Newell-Price

**Affiliations:** Consultant Endocrinologist, Sri Jayewardenepura General Hospital, Colombo 10250, Sri Lanka; Consultant Endocrinologist, Teaching Hospital Kalutara, Kalutara 12000, Sri Lanka; Senior Registrar in Endocrinology, Postgraduate Institute of Medicine, University of Colombo, Colombo 00700, Sri Lanka; Consultant Community Physician, Ministry of Health, Colombo 01000, Sri Lanka; Senior Registrar in Endocrinology, Postgraduate Institute of Medicine, University of Colombo, Colombo 00700, Sri Lanka; Researcher, Department of Molecular Biology and Biotechnology, Postgraduate Institute of Science Peradeniya, Peradeniya 20400, Sri Lanka; Consultant Endocrinologist, Colombo North Teaching Hospital, Ragama 11010, Sri Lanka; Professor of Endocrinology, University of Sheffield, Sheffield S10 2TN, UK

**Keywords:** etomidate, severe Cushing syndrome, Cushing’s disease, hypercortisolism, cortisol

## Abstract

**Background:**

Severe Cushing syndrome is a medical emergency. Etomidate is the only IV option available for treating hypercortisolism, especially in critically ill patients obviating oral medications.

**Methods:**

A systematic review and meta-analysis were conducted on the use of etomidate in the treatment of severe Cushing syndrome. This was registered in PROSPERO, and data reporting was done as per the Preferred Reporting Items for Systematic Reviews and Meta-Analyses guidelines. Thirty-six published articles comprising 76 clinical cases of 78 clinical episodes of etomidate use were included in the analysis for this review.

**Results:**

Etomidate was administered safely to patients with ages ranging from 2 months to 82 years. It served as the first-line treatment in 53.2% of the cases, with 84.3% of patients treated in intensive care unit (ICU) settings. Infusion durations varied from 3 hours to 5.5 months, but 84.8% of treatments were completed in under 2 weeks. Faster cortisol reduction rates were observed in patients with higher baseline cortisol levels (*P* = .02), those receiving a prior bolus dose (*P* = .015), and those given higher initial infusion rates (*P* = .004). Etomidate as first-line therapy (*P* = .01) and in ICU settings (*P* < .01) were associated with more rapid cortisol reduction compared to its use as subsequent therapy or in non-ICU settings. Overall, 80.9% of patients survived to receive definitive treatment.

**Conclusion:**

Etomidate is effective and safe for reducing cortisol levels in Cushing syndrome. There is a need for standardized guidelines on etomidate use, including detailed recommendations for different clinical settings and patient conditions to ensure safety and effectiveness.

Severe Cushing syndrome (SCS) is characterized by elevated serum cortisol levels (>41 µg/dL or 1100 nmol/L, when not due to physiological causes) or a 24-hour urinary free cortisol (UFC) level exceeding 5 times the upper limit of normal, often accompanied by severe hypokalemia (<3.0 mmol/L) and recent-onset complications such as sepsis, opportunistic infections, uncontrolled hypertension, heart failure, uncontrolled hyperglycemia, gastrointestinal hemorrhage, peritonitis with gut perforation, acute psychosis, progressive debilitating myopathy, and thromboembolism [1]. SCS is generally considered very severe when UFC exceeds 10 times the upper limit of normal [2].

SCS poses a challenging medical emergency with high mortality and morbidity rates due to severe hypercortisolism and its associated complications [1, 3, 4]. Immediate therapy and intensive supportive care are crucial to rapidly reduce circulating cortisol levels, sometimes before identifying the underlying cause [3, 4]. Bilateral adrenalectomy may be life-saving in severe cases. Preoperative cortisol reduction with medical therapy has been shown to improve surgical outcomes [4-6].

Guidelines recommend using medical therapy, either alone or in combination, to achieve normalization of cortisol levels, both clinically and biochemically [3]. Available treatments include adrenal steroidogenesis inhibitors such as metyrapone, ketoconazole, fluconazole, mitotane, etomidate, osilodrostat; pituitary-directed therapies for Cushing’s disease (CD) like cabergoline, pasireotide; and glucocorticoid receptor antagonists such as mifepristone, all currently employed in clinical practice [1, 3, 6-9].

Etomidate is the sole IV treatment option available, primarily used in critically ill patients with SCS [3, 10, 11]. Originally developed as an anesthetic induction agent in 1965, etomidate was initially favored for its safety profile, including its hemodynamic stability, cerebral protection, and minimal respiratory depression at therapeutic doses [12-14]. However, concerns arose in 1983 regarding its use in intensive care due to increased mortality in patients receiving prolonged infusions compared to benzodiazepines [15]. Further investigation revealed etomidate's potent inhibition of adrenal steroidogenesis through its action on cytochrome P450-dependent enzymes, particularly 11β-hydroxylase, with lesser effects on other enzymes involved in steroid synthesis [14-20].

Clinical application of etomidate to treat hypercortisolism began in the late 1980s, demonstrating its efficacy in suppressing adrenal function within hours at subhypnotic doses [3, 21]. Recommendations include initial IV administration with a loading dose of 3 to 5 mg, followed by a continuous infusion adjusted to achieve stable serum cortisol levels [3, 23]. Due to its pharmacokinetic properties—75% protein binding, large volume of distribution, primarily renal excretion—lower doses may be required in patients with renal impairment or in the elderly [12, 22, 24, 25]. Etomidate formulations typically include propylene glycol, necessitating central venous administration to minimize local complications [3, 12, 14, 25-27].

Given the ongoing clinical need for effective and safe therapies for SCS, this systematic review evaluates the overall effectiveness and safety of etomidate, aiming to inform and guide clinical practice.

## Methods

### Planning, Registration, and Reporting

Preferred Reporting Items for Systematic Reviews and Meta-Analyses (PRISMA) guidelines were used in reporting the study. The review protocol was registered in PROSPERO with registration number CRD42022343860. The PICO statement was composed as “safety (ie, occurrence of adverse drug reactions) and effectiveness (ie, complete or partial recovery) parameters of etomidate among patients with SCS in studies with or without a comparison group.”

### Literature Search and Article Screening

The literature search was done in EMBASE [28] (Supplementary File 1), PubMed, and Cochrane databases. The primary search was done from the inception of the databases up to December 28, 2021. Relevant keywords and subject headings were used in the search. The following search terms were used to search all databases: high cortisol OR hypercortisolemia OR hypercortisolaemia OR Cushing OR Cushing OR Cushing syndrome OR Cushing disease AND etomidate. The search strategy used in EMBASE and PubMed has been annexed [29] (Supplementary File 2). The reference lists of the selected articles were explored for any potential further studies. Furthermore, a secondary search was done up to February 6, 2022.

The article screening was done in 2 rounds using 3 screening questions: (1) whether the article describes research done on a case(s) of SCS (as defined earlier) treated with etomidate, (2) whether the study presents primary data on use of etomidate, and (3) whether the article includes quantifiable outcomes related to effectiveness or safety.

**Figure 1.**
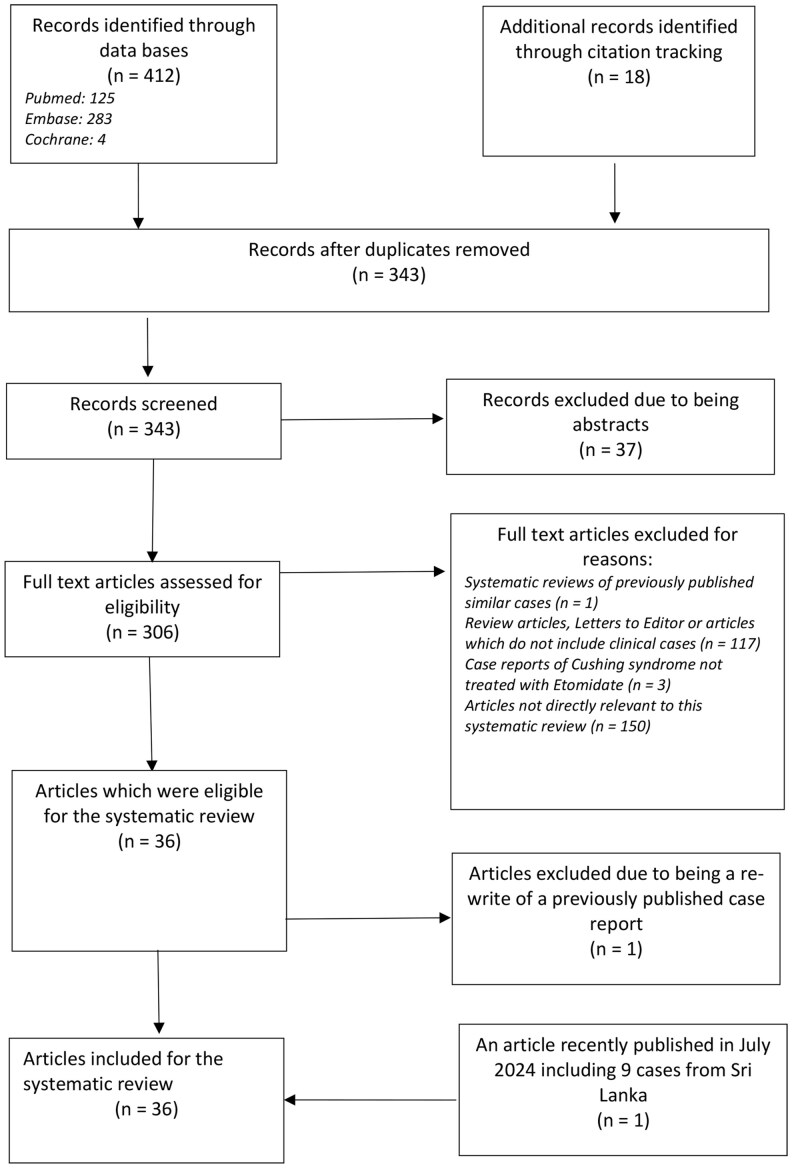
The flow diagram of study selection. This flow diagram shows the stepwise process of article selection and reasons for exclusion of articles prior to finalizing the articles used for the systematic review. There were 283 articles identified from the Embase search, 125 more from the Pubmed search, 4 more from the Cochrane search, and 18 more additional records from citation tracking. One recently published article by the same authors in Sri Lanka was also included. After detailed review and exclusion of articles, 36 articles were used for the systematic review.

Two reviewers independently screened articles in 2 rounds using these 3 eligibility questions. In the first round, screening was done using titles and abstracts. In the second round, complete articles were reviewed. Any discrepancies in study selection were adjudicated by the senior author. The flow diagram of article selection is shown in Fig. 1 and describes the stepwise process of excluding articles not used for the review. One hundred fifty articles were excluded due to not being directly relevant to the study; examples were articles with etomidate used solely as an anesthetic agent in patients without Cushing syndrome, articles in which hypercortisolism did not account for Cushing syndrome, articles on Cushing syndrome with no details on treatment, articles with pituitary adenoma not accounting for CD, and articles not related to Cushing syndrome but an author's name being “Cushing” (n = 1). Following this process, 36 articles were identified, and 2 articles were found to be describing the same content [ie, the case report titled “Continuous Etomidate for the Management of Cushing's Syndrome Complicated by Pulmonary Nocardiosis” by Hays et al published in the *Journal of Pharmacy Practice* was published again in the same year (2021) titled “Use of Continuous Etomidate Infusion to Rapidly Correct Hypercortisolism in a Patient with Disseminated Nocardiosis” by Tasleem et al in *Cereus: Journal of Medical Science*) [30, 31]. Hence a total of 35 articles were eligible for the systematic review. A Sri Lankan case study of 9 cases that was recently published in July 2024 was also included [32].

### Risk of Bias Assessment and Data Extraction Process

It was initially planned to use the Joanna Briggs Institute risk of bias (RoB) assessment tools for case reports and case series, GRADE criteria for any other observational studies or randomized clinical trials, and ROBINS-1 criteria for nonrandomized trials [33, 34]. Since the selected articles included only case reports and case series, the Joanna Briggs Institute tools were used for the RoB assessments. Two reviewers independently completed the RoB assessments, and a third reviewer intervened in any discrepancies.

### Data Extraction and Narrative Synthesis

Data extraction utilized a predefined template comprising 62 items [29] (Supplementary File 2), covering study type, patient characteristics, investigation findings, etomidate dosing regimens with or without other medications, and outcomes.

The cortisol drop rate was computed by dividing the difference between the highest recorded cortisol level immediately before etomidate administration and the lowest recorded cortisol level after infusion initiation by the time taken to achieve this difference in hours. Infusion rates were categorized as “recommended,” “high,” and “low” based on guidelines from the Endocrine Society [3]. Patients treated in intensive care units (ICU) or high dependency units (HDU) were grouped under “ICU setting,” while those treated in regular wards were categorized as “non-ICU setting.” Patients receiving etomidate as a second or subsequent line of treatment were classified under “subsequent line.”

Given the significant clinical and methodological diversity observed, pooled analyses were not pursued. Instead, narrative descriptions were provided for study characteristics, drug regimen, and patient profiles. A narrative synthesis of outcomes was structured across selected domains focusing on effectiveness and safety. Statistical analyses, including chi-square tests, Mann-Whitney U tests, and Kruskal-Wallis tests, were performed using Statistical Package for Social Sciences version 25 to assess differences between groups, considering all available outcome parameters for each case.

## Results

### Study Characteristics

The 36 published articles (28 individual case reports, 7 case series, and 1 case control study) were included [35-37]. Two individual cases were published as a letter to the editor and a scientific letter but included the primary data of patients. These publications were published from 1986 to 2021, and 1 case study was recently published from Sri Lanka in July 2024, written by the same authors. All articles documented the underlying etiology of Cushing's syndrome and reported other patient characteristics [age or age range and sex were documented in 75/76 cases (98.7%); cortisol burden was documented in 74/78 cases (94.9%)].

### Patient Characteristics

This review included a total of 76 (n = 76) patients who received etomidate in 78 clinical episodes. The majority were in the fourth to sixth decades of life with a 1:1 male:female ratio (sex and age were not documented in 1 case among 7 cases, respectively). Table 1 shows the age, sex, and highest basal cortisol level distribution among the participants across the studies. Etomidate was used safely in patients aged 2 months to 82 years with successful outcomes [38, 39].

**Table 1.** Distribution of age, sex, and highest cortisol level among the participants

	Sex			Age category (years)					Highest basal cortisol (nmol/L)				
	M	F	NR	<10	11-30	31-60	>60	NR	<1000	1000-2000	2000-3000	>3000	NR
CD	9	11		2	4	10	1	3	6	10	—	3	1
ECS	19	16		—	5	17	11	2	1	19	4	11	1
ACC	7	7		1	4	4	5		2	9	2	2	
Benign adrenal lesions	2	4	1	2	—	3	—	2	4	2	—	1	—
Total	37	38	1	5	13	34	17	7	13	40	6	17	2
Total	76			76					78				

Abbreviations: ACC, adrenocortical cancer; CD, Cushing's disease; ECS, ectopic ACTH syndrome; NR, not reported.

Multiple complications due to the disease condition were reported: hypertension (n = 37); diabetes (n = 30); hypokalemia (n = 45); metabolic alkalosis (n = 15); myopathy (n = 27); psychosis and mental illness (n = 20); infection/sepsis (n = 36); thrombosis (n = 6) [40]; fractures (n = 8); and other rare complications such as cerebral hemorrhage, hypertensive encephalopathy, and cardiac involvement.

### Indications for Etomidate Therapy

Etomidate was primarily administered to manage hypercortisolism in 98.7% of cases (77 out of 78), with secondary uses including psychosis control in 10 cases and anesthesia induction in 1 case reported in 1986, where cortisol reduction was an observed secondary effect [23].

Among the cases reviewed, etomidate was utilized as a first-line agent in 53.2% (33/62), as a second-line treatment in 32.2% (20/62), and as a subsequent option in 14.5% (9/62). It was often chosen as the initial treatment when patients were unable to take oral medications due to reasons such as mechanical ventilation (4 cases), psychosis (7 cases), severe abdominal sepsis (3 cases), renal dysfunction (2 cases), lack of availability of other medications (1 case), severe complications with organ dysfunction (5 cases), or participation in a research protocol (12 cases). In cases where oral medications were no longer feasible, etomidate was used as a second-line or subsequent treatment due to factors like ventilation during other therapies (11 cases), psychosis leading to oral medication refusal (6 cases), gut perforation with sepsis (2 cases), failure to achieve cortisol normalization with previous therapies (15 cases), or intolerance and side effects including liver dysfunction (10 cases).

Etomidate was administered alone in 50/62 patients and in combination with other drugs in the remaining 10 cases (ketoconazole in 10 cases, metyrapone in 1 case, and mifepristone in 1 case). It was used despite the presence of organ dysfunction such as renal failure (8 cases), liver dysfunction (11 cases), cardiac failure (3 cases), and respiratory failure (3 cases).

### Etomidate Regime

#### Setting: ICU vs non-ICU

The majority of patients (84.3%, n = 54/64) were treated in an ICU (n = 53)/HDU (n = 1) and 3 were treated in the ward setting with close monitoring [38]. There were 10 cases that described the initiation of etomidate in a non-ICU setting, later transferring to an ICU setting. ICU was selected as the setting in a majority of the cases due to life-threatening disease requiring ventilation. Thirteen cases that initiated or continued etomidate in a non-ICU setting were from 2 case series; 10/13 were from the study published in 2020 comparing etomidate use in an ICU vs non-ICU setting [38].

#### Duration of the infusion

The duration of etomidate infusion varied widely, ranging from 3 hours (for anesthesia induction) to 5.5 months (for palliative care). Excluding these extreme cases, the average duration of infusion was 8.74 days, with durations spanning from 8 hours to 58 days. Etomidate was used for less than 2 weeks in the majority of cases, accounting for 84.8% (56 out of 66), while it was administered for longer durations in 10 cases: 2 to 4 weeks in 5 cases and more than 4 weeks in 5 cases [41].

Patients with malignancies, specifically adrenal cortical carcinoma (ACC), ectopic ACTH-dependent Cushing's syndrome (EAS), and CD, had longer average infusion durations: 7.33 days for ACC (14 cases), 11.92 days for EAS (27 cases), and 6.63 days for CD (12 cases). Patients with benign adenomas or hyperplasia received etomidate for an average of 3.86 days (5 cases).

#### Dosage regimes

##### Administration of a bolus prior to infusion

Etomidate boluses were administered in 24 cases, followed by infusions starting at various rates: low (12 cases), recommended (5 cases), and high (7 cases). A bolus dose was given in only 1 case outside of an ICU setting (2.5 mg). Of these, 15 out of 24 patients received the bolus according to Caroll et al's protocol [42]. The bolus doses were given as 0.3 mg/kg (9 cases), 2.5 mg (3 cases), 5 mg (9 cases), 10 mg (1 case), and 0.05 mg/kg (1 case). The average baseline serum cortisol level was 2486 nmol/L in the bolus group and 1976 nmol/L in the group that did not receive a bolus.

##### Etomidate infusion rate

Infusion rates were documented in 72 cases, with 39 cases (54%) adhering to guideline-recommended doses [2]. The most frequently used starting infusion rates were 0.03 mg/kg/hr (10 cases), 0.04 mg/kg/hr (4 cases), and 2.5 mg/hr (11 cases). Fourteen cases started with high doses, and 19 cases began with low doses, with uptitration reported in 7 cases. Low starting rates included 0.02 mg/kg/hr (14 cases, 3 uptitrated), 2 mg/hr (3 cases, 2 uptitrated), 1 mg/hr (1 case), and 1.25 mg/hr (2 cases, both uptitrated). In 12 out of 19 cases, a low infusion rate followed an initial bolus.

The highest reported infusion rate was 6 mg/kg/hr (0.1 mg/kg/min), with other commonly used higher doses including 0.3 mg/kg/hr (9 cases), 8 mg/hr, 15 mg/hr, and 16 mg/hr. High doses were administered according to Caroll et al's protocol (9 cases), in cases of high basal serum cortisol (>3000 mmol/L, 2 cases), for anesthesia induction (1 case), and for psychosis control (1 case) [42]. Krakoff et al documented a case where etomidate was used for 5.5 months at varying rates, including high rates during surgery and low-rate prolonged infusion during renal failure [43]. Except for 3 cases published in 2020, high rates were utilized before the publication of the Endocrine Society guidelines in 2015 [3, 38].

In cases not treated in ICU settings, Endocrine Society clinical practice guideline-recommended doses or lower starting doses (0.02 mg/kg/hr to 0.04 mg/kg/hr or 2-3 mg/hr) were predominantly used [3]. Infusion rates were safely uptitrated to 0.08 mg/kg/hr and 5 mg/hr in ward settings without reported side effects, continuing for durations ranging from 8 to 58 days.

Though most cases had not specifically described how the etomidate infusion was diluted and prepared, Carroll et al described 9 cases of diluted 2 mg/mL of Etomidate Lipuro solution in a 1:1 ratio with 5% dextrose in water forming a 1 mg/mL concentration [42].

##### Use of block and replacement strategy

Eighteen cases utilized a block and replacement strategy. Among them, 3 cases had basal serum cortisol levels below 1000 nmol/L, 10 between 1000 and 2000 nmol/L, and 4 exceeding 3000 nmol/L. All patients receiving block and replacement therapy were treated in ICU settings, achieving the lowest serum cortisol values ranging from 140 to 463 nmol/L through infusion titration. The outliers of this lowest cortisol range were a patient whose lowest cortisol was 36 nmol/L prior to adding the hydrocortisone infusion and a patient whose lowest cortisol was recorded as 4110 nmol/L, a significant drop from the baseline cortisol, which was 13 000 nmol/L. The lowest recorded cortisol level was recorded before starting steroids in 12 cases and after starting steroids in 3 cases, and the timing of the lowest recorded cortisol was not recorded in 3 cases.

An etomidate bolus was administered in only 1 case employing block and replacement therapy [31]. Steroid replacement was administered alongside etomidate infusion at low rates (1 case), high rates (4 cases), and recommended rates (12 cases). The duration of block and replacement comprising the combination of etomidate and steroids ranged from 72 hours to 2.7 months (1920 hours). Initiation of steroids as part of the block and replacement regime after commencing etomidate ranged from 30 hours to 7 weeks. In the case where etomidate was administered for 5.5 months, concomitant steroid replacement was introduced 7 weeks after commencing etomidate and continued for 2.7 months [43]. Hydrocortisone was used as the steroid in 7 cases and dexamethasone in 2 cases. Steroids were administered as bolus doses in 5 cases and as infusion in 6 cases, while the other cases did not specify the steroid regime.

##### Monitoring during treatment

All patients received close monitoring by expert teams, with assessments conducted every 4 to 8 hours, including frequent serial cortisol level measurements to guide dose titration. Sedative scores were mentioned in only 13 cases. Clinical and biochemical parameters such as blood pressure, serum electrolytes, cortisol, UFC, plasma ACTH, serum 11-deoxycortisol, testosterone, dihydroepiandrosteniodione sulphate, androstenedione, 17 hydroxyprogesterone, and blood sugar were monitored using immunoassay and mass spectrometry techniques. Expert teams ensured rigorous monitoring throughout the treatment, with cortisol levels specifically assessed every 4 to 8 hours.

##### Type of etomidate used

Propylene glycol (PG) was the commonly used preparation (n = 26), while Lipuro was reported in 11 cases.

### Effectiveness of Etomidate Therapy

#### Cortisol-lowering efficacy of therapy

Among those with a baseline cortisol level >3000 nmol/L (17 patients), 4 had their lowest cortisol remaining above 1000 nmol/L with a drop of 60% to 75%. These cases included individuals with EAS (3 cases) and ACC (1 case) complicated by sepsis and perforated bowel (2 cases), where cortisol levels above 1000 nmol/L were deemed acceptable despite treatment. Notably, 2 patients in this group died while receiving etomidate, potentially influencing the achieved cortisol levels. The cause of death in these 2 patients was metastatic cancer with perforated diverticulitis in 1 case and sepsis, renal failure, and disseminated intravascular coagulation in the other case [32, 42]. Etomidate was used as first-line treatment in 2 cases, with 1 patient receiving a bolus followed by a low-rate infusion.

Four out of 17 patients achieved the lowest cortisol level between 850 and 1000 nmol/L with a drop of 70% to 80%, while 9 out of 17 achieved cortisol levels below 850 nmol/L with drops ranging from 82% to 97%, including cases of ECS (7 cases), CD (1 case), and ACC (1 case).

Among the 43 cases with baseline cortisol levels between 1000 and 3000 nmol/L, all achieved the lowest cortisol levels below 1000 nmol/L. Thirty out of 43 patients (CD, 8; ACC, 7; ECS, 14; adrenal adenoma, 1) reached cortisol levels below 450 nmol/L with drops ranging from 69% to 100%, while 11 out of 43 cases (CD, 2; ACC, 3; ECS, 5; adrenal adenoma, 1) lowered cortisol to 450 to 850 nmol/L with drops ranging from 49% to 76%. All 12 cases with baseline cortisol levels below 1000 nmol/L achieved the lowest cortisol levels below 450 nmol/L with drops ranging from 44-81%.

In malignancy cases (ECS/ACC), 27 out of 47 patients achieved cortisol levels <450 nmol/L, with varying results in other ranges: 13, 3, and 4 cases achieved the lowest cortisol levels of 450-850 nmol/L, 850-1000 nmol/L, and above 1000 nmol/L respectively. Among benign cases (CD and adrenal adenoma), 18 out of 25 achieved cortisol levels <450 nmol/L.

Etomidate administration showed varied outcomes based on bolus use and infusion rates: 11 cases achieved cortisol levels below 450 nmol/L with bolus, while 10 achieved 450 to 850 nmol/L, and higher levels were noted in a few cases. High infusion rates in 14 cases led to cortisol levels below 450 nmol/L in 12 instances with a 76% to 97% cortisol drop, while low infusion rates in 20 cases resulted in 8 achieving cortisol levels below 450 nmol/L. Out of 13 patients treated in the non-ICU setting, 7 achieved cortisol levels <450 nmol/L.

**Figure 2.**
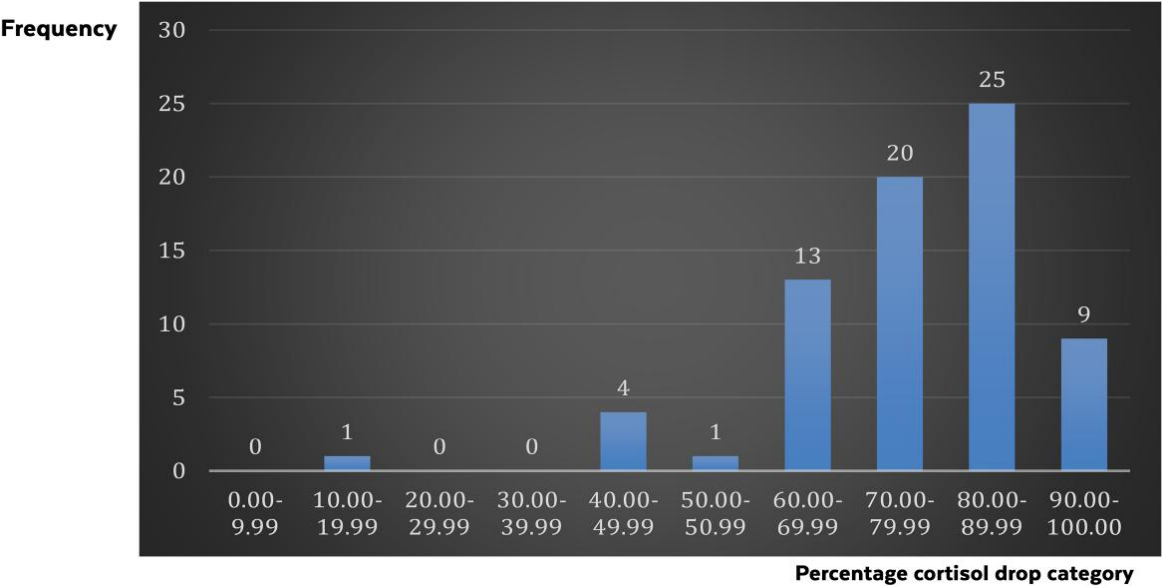
Distribution of percentage of cortisol drop (n = 73). This figure shows the frequencies of cases in each of the percentage of cortisol drop categories. The categories of cortisol drop percentage included 10 categories with a range of 10% in each category. Most cases had a cortisol drop from etomidate ranging from 80% to 89.99%.

Figure 2 illustrates the distribution of initial cortisol levels, the magnitude of drop, and the lowest achieved cortisol levels.

#### Rate of cortisol drop

The rate of serum cortisol drop ranged widely from 3.69 to 224.82 nmol/L/hr. Cases with lower baseline serum cortisol levels experienced slower cortisol reduction rates, while those with higher baseline levels showed faster reductions, a statistically significant finding (*P* = .02). There was no significant difference in cortisol reduction rates across various causes of SCS (*P* = .743). (Table 2)

**Table 2.** Treatment outcomes

		Median time to achieve documented lowest cortisol in hours (IQR)	*P*-value	Median rate of cortisol drop nmol/L/hour (IQR)	*P*-value	Survival to definitive therapy (n)	*P*-value
Etiology of SCS	CD (n = 20)	25 (11.75-64.50) (missing =2)	.153	28 (16.75-75.5) (missing = 2)	.743	12/13 (missing = 7)	.784*^b^*
	Benign adrenal lesions (n = 7)	24 (9.0-36.00) (missing = 1)		65 (17.00-152.5) (missing = 1)		4/5 (missing = 2)	
	ACC (n = 15)	48 (24.00-86.75) (missing = 1)		29 (12.50-43.25) (missing = 1)		12/15 (missing = 0)	
	ECS (n = 36)	48 (24.00-95.50) (missing =8)		37 (12.50-107) (missing = 8)		23/30 (missing = 6)	
Baseline cortisol (NR = 2)	<1000 (n = 13)	24 (24.0-62.0) (missing =2)	.593	20 (11.0-23.0) (missing = 2)	.02*^a^*	9/9 (missing = 4)	.317*^b^*
	1000-1999 (n = 40)	48 (14.25-72.0) (missing = 4)		29.5 (11.5-94.0) (missing = 4)		25/31 (missing = 9)	
	2000-2999 (n = 6)	56 (24.0-118.5) (missing = 0)		42 (20.75-97.5) (missing = 0)		4/5 (missing = 1)	
	>3000 (n = 17)	43 (22.0-132.5) (missing = 4)		68 (42.0 to139.5) (missing = 4)		11/16 (missing = 1)	
Administration of bolus (NR = 6)	Bolus given (n = 24)	29 (11.00-94.00) (missing = 1)	.208	56 (23.0-107.0) (missing = 1)	.015*^a^*	15/18 (missing = 6)	.466*^b^*
	Not given (n = 48)	48 (24.00-72.00) (missing = 6)		24 (13.00-52.00) (missing = 6)		32/41 (missing = 7)	
Starting infusion rate (NR = 6)	High rate (n = 14)	11 (11.00-24.00) (missing = 1)	<.01*^a^*	107 (46.5-107.0) (missing = 1)	.004*^a^*	6/7 (missing = 7)	.816*^b^*
	Recommended rate (n = 38)	43 (24.00-67.00) (missing = 5)		21 (10.00-60.00) (missing = 5)		26/33 (missing = 7)	
	Low rate (n = 20)	72 (33.00-134.0) (missing = 1)		28 (18.00-44.00) (missing = 1)		15/19 (missing = 1)	
Line of therapy (NR = 16)	First line (n = 33)	24 (11.00-35.25) (missing = 5)	<.01*^a^*	62.5(23.5-107.0) (missing = 5)	.04*^a^*	16/18 (missing = 15)	.166*^b^*
	Subsequent therapy (n = 29)	60 (40.00-96.00) (missing = 6)		18 (8.00-55.0) (missing = 6)		21/29 (missing = 0)	
Setting (NR = 12)	ICU (n = 53)	33 (20.00-61.0) (missing = 8)	<.001*^a^*	40 (20.0-107.0) (missing = 8)	.003*^a^*	34/44 (missing = 9)	.444*^b^*
	Non-ICU (n = 13)	108 (54.0-192.0) (missing = 1)		15.5 (7.25-27.0) (missing = 1)		11/13 (missing = 0)	

Abbreviations: ACC, adrenal cortical carcinoma; CD, Cushing’s disease; ECS, ectopic ACTH syndrome; ICU, intensive care unit; IQR, interquartile range; NR, not reported; SCS, severe Cushing syndrome.

^*a*^Statistically significant difference.

^*b*^Exact significance (2-sided).

Patients who received a prior etomidate bolus exhibited a faster rate of cortisol reduction compared to those without a bolus (*P* = .015). High initial infusion rates of etomidate led to a quicker cortisol reduction compared to guideline-recommended or low starting rates (*P* = .004). (Table 2).

The rate of cortisol reduction was significantly faster when etomidate was used as first-line therapy compared to subsequent therapy (*P* = .04). Additionally, cortisol reduction rates were notably higher in ICU settings compared to non-ICU settings (*P* = .003) (Table 2).

#### Time to achieve lowest documented cortisol

The time taken to achieve the lowest documented serum cortisol level ranged from 3 to 264 hours (median 38 hours, mean 60.22 hours). In 9 cases, cortisol reached the desired lowest levels in less than 12 hours; 7 out of these 9 cases were treated with a high starting infusion rate of etomidate. Conversely, 10 cases took more than 96 hours to achieve the lowest cortisol levels; among these, 9 had a malignant etiology (6 ECS, 3 ACC) and 4 had a baseline cortisol level exceeding 3000 nmol/L, all treated with either low (6 cases) or recommended starting infusion rates of etomidate.

Patients with higher baseline serum cortisol levels generally took longer to reach the lowest recorded serum cortisol level, although this difference was not statistically significant (*P* = .593). There were no significant differences observed in the time required to achieve the lowest cortisol level across different etiologies of SCS or baseline cortisol level categories or whether a prior etomidate bolus was administered or not (*P* > .05). However, patients treated with a higher starting infusion rate of etomidate (*P* < .01), those receiving etomidate as first-line therapy (*P* < .01), and those managed in the ICU setting (*P* < .01) required less time to achieve the desired lowest cortisol level. (Table 2).

#### Survival

Fifty-one out of 63 patients (80.9%, 15 not reported) were reported to have survived and underwent definitive treatment for the etiology (n = 39; surgery, n = 18; bilateral adrenalectomy, n = 7; ECS tumor resection, n = 3; palliative surgery, n = 6; transsphenoidal adenectomy, n = 6; unilateral adrenalectomy,n = 8; medical and oncological treatment n = 3) [44].

**Figure 3.**
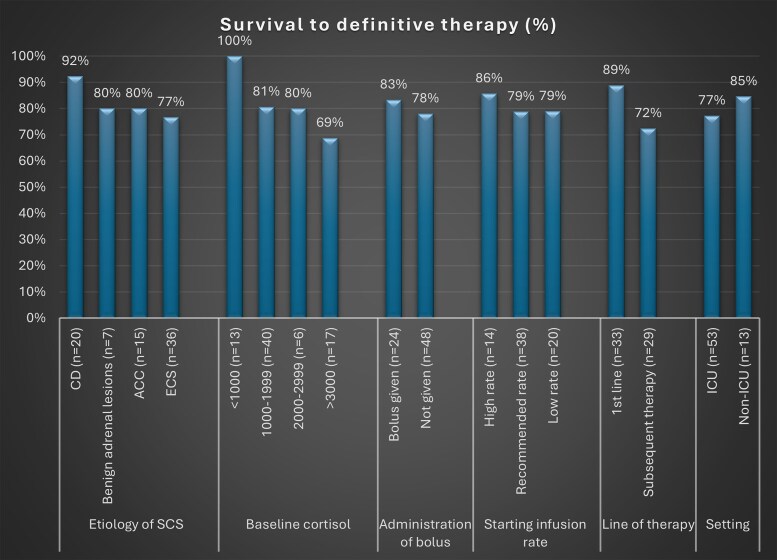
The distribution of survived percentage until definitive therapy between different groups. This bar chart shows the percentage of cases that survived up to definitive treatment, categorized based on etiology of severe Cushing syndrome, baseline cortisol, administration of prior bolus of etomidate or not, starting infusion rate of etomidate, line of treatment with etomidate (first line or subsequent line of therapy), and treatment setting (ICU or non-ICU). Abbreviation: ICU, intensive care unit.

Documented overall mortality in the series was 43.6% (n = 24/55), while only 12/63 (19%) died before the next line of treatment (3 ACC, 8 ECS, 1 bilateral adrenal hyperplasia, 1 CD) (Fig. 3).

The youngest patient who died before definitive treatment was a 14-month-old with bilateral adrenal hyperplasia, complicated by hypertension [45]. Ten patients over 40 years old had malignancies (ACC or ECS) and severe complications from Cushing's syndrome, such as cardiac issues, cerebral problems, sepsis-related complications, or liver dysfunction. All had baseline serum cortisol levels >1000 nmol/L, with 5 exceeding 3000 nmol/L. Ten were managed in the ICU/HDU due to severe complications, while 2 were in the ward for palliative care despite severe disease.

Eight patients died during etomidate infusion. Three received a prior bolus, and 2 started with a high infusion rate; all others, including those who died during infusion, received etomidate at low or recommended rates. A block and replacement regimen was used in 2 cases with all but 1 maintaining cortisol levels above 450 nmol/L; in this exceptional case, the lowest cortisol recorded was 100 nmol/L. Twelve patients died after definitive therapy. Statistically significant differences in survival until definitive therapy were not observed among the comparison groups detailed in Table 2 (*P* > .05).

### Safety and Side Effects

#### Safety

Etomidate was administered to 8 patients under 20 years old, including 6 patients under 16 years. Details of 6 pediatric patients (age < 16 years) aged 2 months, 14 months, 6 years (3 cases), and 14.7 years are included in Table 3. All pediatric patients were treated with etomidate exclusively in the ICU setting, and etomidate was continued for a total of less than 2 weeks in those who had that information. A weight-based infusion rate of 0.03 mg/kg/hr was used in all pediatric cases except 1 who followed a fixed rate of 3 mg/kg, the usual recommended rate for adults. One pediatric patient aged 2 months old received a prior etomidate bolus (0.3 mg/kg), and others did not. The 14-month-old patient died during etomidate infusion, and the 2-month-old died after definitive surgery, while the others survived successfully [45, 46] (Table 3).

**Table 3.** Details of the pediatric cases with SCS treated with etomidate

Reference	Kwon et al (2022) [46] [South Korea]	Song et al (2020) [47] [South Korea]	Yun Castilla et al (2017) [45] [Spain]	Chan et al (2011) [48] [UK]	Mettauer et al (2009) [49] [UK]	Greening et al (2005) [39] [UK]
Age	2 months	6 years	14 months	14.7 years	6 years	6.2 years
Sex	Female	Male	Female	Female	Male	Male
Etiology of SCS	Adrenocortical carcinoma	Unilateral adrenal adenoma	Bilateral adrenocortical nodular hyperplasia	Cushing disease	Cushing disease	Cushing disease
Indication for etomidate	NR*	NR*	Not responding to other therapies	Control of psychosis, refusal of oral intake	Ventilated and inability to take orally, liver dysfunction	Liver dysfunction, not responding to other therapies
Etomidate line of therapy	First	First	Second	NR*	Third	Third
Treatment setting	NR*	ICU	ICU	ICU	ICU	ICU
Prior bolus given or not	Yes (0.3 mg/kg)	No	No	No	No	No
Starting infusion rate	0.03 mg/kg/hr	0.03 mg/kg/hr	0.03 mg/kg/hr	3 mg/hr	0.03 mg/kg/hr	0.03 mg/kg/hr
Block and replacement regime	NR*	Yes	Yes	Yes	NR*	Yes
Baseline cortisol prior to etomidate (nmol/L)	1875	728	4270	986	1200	1250
Lowest achieved cortisol (nmol/L)	599	152	852	186	200	191
Percentage of cortisol drop	68.1	79.1	80.0	81.1	83.3	84.7
Time to eucortisolemia (hours)	8	NR*	24	62	48	57.6
Rate of cortisol drop (nmol/L/hour)	159.5	NR	142.4	12.9	20.8	18.4
Duration of etomidate infusion (days)	0.3	NR*	5	NR*	12	12
Definitive treatment	Chemotherapy	Unilateral adrenalectomy	None (died during infusion)	Bilateral adrenalectomy	Bilateral adrenalectomy	Bilateral adrenalectomy
Overall mortality	Died (later)	Survived	Died (during infusion)	Survived	Survived	Survived

Abbreviations: ICU, intensive care unit; NR*, not reported; SCS, severe Cushing syndrome.

In the elderly group (aged >70 years), etomidate was administered in both ICU and non-ICU settings with varied dosage regimens. Some elderly patients who received etomidate at a high rate experienced somnolence, and 2 deaths occurred during infusion. Ten patients with renal impairment received etomidate safely without adverse effects related to etomidate or propylene glycol (PG preparation used in 6 cases). A prior etomidate bolus was administered in some cases, with 2 out of 10 using a high starting dose rate, while others used low or standard rates. Eight out of 10 patients achieved serum cortisol levels <450 nmol/L with etomidate, and all reached their lowest cortisol levels within 11 to 48 hours. Krakoff et al reported the use of etomidate in a patient with acute renal failure undergoing haemodialysis, showing continued cortisol level response [43]. Liver dysfunction caused by SCS or other therapies was considered an indication for initiating etomidate therapy.

#### Side effects

Five reported somnolence, and none received a prior etomidate bolus [38]. One patient experienced somnolence with a recommended starting infusion rate of 0.03 to 0.05 mg/kg/hr over 20 days in a ward, achieving target cortisol levels in 192 hours [38]. The remaining 4 received a high starting infusion rate (0.1-0.3 mg/kg/hr) in the ICU for 1 to 7 days, achieving desired cortisol levels within 24 hours [38]. Despite somnolence, all patients survived and continued therapy.

Sedation occurred in 3 cases: 2 under higher anesthesia doses and 1 with psychosis [35, 50, 51]. Detailed data were available for 2 cases where high starting infusion rates achieved desired cortisol levels in 3 and 8 hours. Two cases reported nausea, vomiting, and lightheadedness [38]. Myoclonus was noted in a case with 5.5 months of etomidate use [43]. Among 26 who received PG preparation of etomidate, only 2 developed PG related side effects including hemolysis in 1 case and increased serum osmolality in the other case [52, 53]. Despite multiorgan dysfunction, etomidate was continued without worsening [15].

## Discussion

This is the first documented systematic review on the overall effectiveness and safety of IV etomidate in SCS, and it fills an evidence gap on the management of SCS. The main findings reflect that etomidate is safe and effective for this indication.

### Clinical Context of Etomidate Use

Since its introduction in the 1960s, etomidate took nearly 2 decades to gain recognition as an adrenostatic agent for managing hypercortisolism [3, 12-14, 21]. Early literature suggested it might be more effective in ACTH-independent diseases or those with a fixed ACTH drive compared to CD [54]. However, our review did not find significant differences based on etiology. In elderly patients, etomidate should be used cautiously, preferably starting with low infusion rates and closely monitoring its effects.

Etomidate has demonstrated efficacy across various degrees of hypercortisolism. It is reasonable to expect that higher baseline cortisol levels may require longer to normalize. In urgent situations, high infusion rates can be considered with careful monitoring to rapidly lower elevated cortisol levels, provided these doses do not induce sedation unless the patient is ventilated.

### Indications and Place of Etomidate vs Other Oral Therapies in SCS

While lowering cortisol as its primary effect, its ability to control acute psychosis and induce and maintain anesthesia during surgery is an advantage of using this medication over other therapies in the presence of such indications. The inability to administer oral agents such as in ventilated patients or in the presence of acute psychosis, abdominal sepsis precluding oral intake was the main indication for etomidate in SCS as first-line therapy in the review, as etomidate is the only available IV agent for treating hypercortisolism. Etomidate was used as a subsequent option in the presence of adverse effects or poor response to other cortisol-lowering therapies. The degree of cortisol elevation was not considered a main indication to choose etomidate over other therapies. It was used as a combination therapy where the initial response was poor with other oral agents alone. There was no significant difference in outcome with regard to undergoing definitive treatment and short-term mortality depending on whether etomidate was given as first line, second line, or subsequently or whether it was given alone or in combination. This shows the versatility of this agent in any clinical scenario, enabling the clinician to adapt to any situation based on personal expertise and resources.

Clinicians may opt for oral agents in SCS due to the ease of administration without close monitoring, but these orally administered agents have their own drawbacks. Ketoconazole can cause hepatotoxicity, metyrapone can cause hirsutism with long-term use, and mifepristone can cause hypertension and gastrointestinal intolerance [55-57]. Some of these agents may not be licensed for the use of Cushing syndrome in some countries due to the cost considerations. The newer drugs such as osilodrostat may not be available or affordable in resource-poor settings [6].

Reasons for the majority of cases not using etomidate as a first-line agent may be the lack of clear guidelines for safe use of etomidate in different clinical scenarios and inadequate intensive monitoring facilities despite the wide availability of the drug even in resource-poor settings. The risk of adrenal insufficiency and sedation can be avoided by close monitoring and careful titration. Considering the current practice, optimal monitoring in an intensive care facility is considered the best option, but our analysis showed that etomidate can also be used safely in the non-ICU setting provided close monitoring is facilitated. If etomidate is introduced safely in the non-ICU setting, this can avoid the hidden cost to the health sector from providing ICU care even in settings where free health care is provided. These findings were promising for increasing indications for use of etomidate in non-ICU settings. The monitoring requirement is less intensive with low-dose infusions, and this evidence has been shown to be noninferior to the standard doses with or without the initial bolus.

This evidence points to the need to formulate and validate standard universal guidelines for the safe use of etomidate in different clinical contexts and settings and for guidance for the most frequent use as a first-line agent.

### Etomidate Regime and Its Efficacy

The common use of the PG preparation of etomidate was likely due to it being the formulation that was initially available in practice, though the lipid formulation is now widely available. Etomidate compounds containing PG as the solubilizer (eg, Hypnomidate®, JANSSEN-CILAG, Neuss, Germany) are known to cause injection-related pain, venous sequelae, and rarely hemolysis, which are thought to be due to the unphysiologically high osmolality of PG [25, 26]. However, these adverse effects were mostly avoided provided the PG level did not reach toxic levels. The etomidate formulation based on a lipid emulsion as the carrier (Etomidat-® Lipuro, B. Braun, Melsungen, Germany) induces nearly no side effects [26, 58].

In 2019 Carroll et al published a study on validation of a standard protocol for etomidate infusion for the management of SCS in which an institutional etomidate protocol was used to safely reduce cortisol levels until more long-term medical or definitive surgical therapy could be instituted [42]. Constantinescu et al in 2020 showed that etomidate used in the non-ICU setting with low starting infusion rates was safe and noninferior to its use in the ICU at higher starting infusion rates, and our analysis confirmed the same observation [38]. This evidence can be used in formulating universal guidelines for etomidate use.

Bolus administration resulted in a rapid cortisol drop over a shorter time period without significant side effects. Therefore, a bolus could be given if rapid normalization is required; however, it is prudent to use it cautiously in non-ICU settings until further evidence is available. Based on the review, a 2.5 to 5 mg or weight-based (0.03 mg/kg) single bolus can be used followed by a low-dose infusion rate, which can be uptitrated as needed.

In the present review, most of the etomidate regimes used complied with the standard recommendations. Higher doses had become seemingly unpopular since the publishing of recommendations on etomidate administration in the *Journal of Clinical Endocrinology and Metabolism* in 2015 [3]. Lower and recommended doses were as effective as higher doses in achieving the desired cortisol drop within a reasonable time period [59, 60]. The use of high-rate infusions in emergency settings especially during surgery and in psychosis was successful where sedation was an expected outcome [61]. If a high dose is used in a non-ICU setting, caution should be taken to monitor for sedation, and cortisol should be frequently assessed to detect rapid normalization of cortisol [61, 62].

A block and replacement regime was implemented when the cortisol level dropped significantly in anticipation of impending adrenal suppression, as most of the patients in this category showed lower cortisol trough values. The majority received IV or oral hydrocortisone whereas a few received dexamethasone. Steroid replacement in etomidate treatment is not mandatory even in the non-ICU setting but could be beneficial in settings where frequent cortisol monitoring is not practical.

### Monitoring and Safety Profile of Etomidate

The Endocrine Society guidelines recommend monitoring the level of sedation using standardized sedation scores and cardiovascular and metabolic parameters [3]. This systematic review showed minimal development of adverse effects of etomidate, with sedation and somnolence being the most common. Biochemical monitoring of the control of hypercotisolemia is straightforward with frequent measuring of cortisol levels. However, as illustrated in the management of severe CD and MRSA bacteremia by Wong et al in 2019, the accumulated precursors of cortisol could cross-react with standard cortisol immunoassays unless mass spectrometry is used [62]. Metyrapone and Osilodrostat, which are hypocortisolaemic agents, also have the limitation of cross-reactivity with cortisol levels unless mass spectrometry is used. In resource-poor settings where mass spectrometry is not freely available, especially when frequent monitoring of cortisol levels every 4 to 8 hours is required, etomidate is a better treatment option than metyrapone.

Etomidate has a safe cardiovascular and metabolic profile. Mifepristone can cause severe hypokalemia and hypertension in addition to hypokalemia and hypertension already present in SCS. Etomidate is a good alternate treatment in patients who have preexisting cardiovascular and metabolic derangement due to SCS. It has a relatively quick onset and offset of action, and its easy titratability makes it an ideal agent for treating SCS. Etomidate can be used across a wide range of time durations ranging from transient use in acute severe hypercortisolism to long-term maintenance when other drugs are not an option.

### Outcome of SCS Treated With Etomidate

Irrespective of the patient and disease characteristics, in almost all cases, the desired cortisol drop was achieved over reasonable time periods. In general, it can be expected that cortisol will normalize within 12 to 96 hours with the aforementioned, commonly used etomidate regimes.

Etomidate was proven to be successful in achieving short-term survival in SCS. Preoperative normalization of cortisol within a desirable time period facilitated successful surgical outcomes and overall survival, while it also reduced morbidity and mortality prior to definitive treatment. Etomidate could be successfully used in palliative settings as well, in combination with chemotherapy, immunotherapy, and other medical therapies as bridging or long-term therapy in settings with facilities for IV drug administration such as in hospice [55, 63].

### Limitations and Strengths of the Review

This review identified several limitations that impact the comprehensive analysis of data across cases. One major issue was the lack of uniformity among cases, which complicates pooled data analysis despite reflecting real-world practice. It is worth noting that this review is currently the only one to assess the efficacy and safety profiles of various etomidate dosage regimens in treating SCS. Although abstracts were excluded, complete articles sometimes lacked crucial information regarding the specific etomidate regimen and treatment outcomes. Despite these shortcomings, these articles were still included in the analysis due to their relevance and the limited number of publications available. Therefore, there is a clear need for more case reports and case series to further characterize etomidate's role in treating SCS across diverse settings.

In comparing infusion rates, doses were categorized based on initial infusion rates, with subsequent titrations not always documented, which may affect the statistical significance of findings. An important biochemical consideration is that etomidate use can elevate 11-deoxycortisol levels, the immediate precursor of cortisol, due to its inhibition of 11-β-hydroxylase. This inhibition can lead to cross-reactivity in current cortisol immune assays, potentially overestimating true cortisol concentrations. Some studies in this review highlighted this issue, recommending the use of mass spectrometry to mitigate assay interference. Consequently, the analysis of cortisol values in this study may be somewhat flawed, as standard assays predominated across most cases.

## Conclusion and Recommendations

Etomidate is versatile in treating Cushing syndrome across all etiologies, regardless of cortisol levels or complications. It can be employed as any line of therapy, either alone or in combination. Etomidate effectively lowers cortisol levels rapidly and safely, without causing adrenal insufficiency when closely monitored. Higher infusion rates of etomidate initiate faster cortisol reduction, but dosing should be tailored based on treatment setting, renal function, and patient characteristics. A prior bolus of etomidate enhances cortisol reduction but requires subsequent low-rate infusion. In settings lacking intensive care or when ventilation is not necessary, etomidate can be safely administered outside intensive care settings with vigilant monitoring by expert teams, utilizing low initial infusion rates without a bolus.

This review underscores the necessity of developing and validating universally standardized guidelines for the safe and effective utilization of etomidate. These guidelines should include distinct recommendations tailored to various clinical scenarios, such as treatment settings (ICU and non-ICU), renal impairment, and other comorbidities. Specifically, the guidelines should delineate indications for employing low vs high infusion rates, prior bolus administration, and the use of block and replacement regimens.

## Disclosures

The authors have nothing to disclose.

## Data Availability

Original data generated and analyzed during this study are included in this published article or in the data repositories listed in References.

## References

[1] Alexandraki KI, Grossman AB. Current strategies for the treatment of severe Cushing's syndrome. Expert Rev Endocrinol Metab. 2016;11(1):65‐79. 30063449 10.1586/17446651.2016.1123615

[2] Lavoillotte J, Mohammedi K, Salenave S, et al Personalized noninvasive diagnostic algorithms based on urinary free cortisol in ACTH-dependant Cushing's syndrome. J Clin Endocrinol Metab. 2024;109(11):2882‐2891. 38609171 10.1210/clinem/dgae258

[3] Nieman LK, Biller BM, Findling JW, et al Treatment of Cushing's syndrome: an endocrine society clinical practice guideline. J Clin Endocrinol Metab. 2015;100(8):2807‐2831. 26222757 10.1210/jc.2015-1818 PMC4525003

[4] van den Bosch OF, Stades AM, Zelissen PM. Increased long-term remission after adequate medical cortisol suppression therapy as presurgical treatment in Cushing's disease. Clin Endocrinol (Oxf). 2014;80(2):184‐190. 23841642 10.1111/cen.12286

[5] Puglisi S, Perotti P, Barbot M, et al Preoperative treatment with metyrapone in patients with Cushing's syndrome due to adrenal adenoma: a pilot prospective study. Endocr Connect. 2018;7(11):1227‐1235. 30352400 10.1530/EC-18-0400 PMC6215797

[6] Hinojosa-Amaya JM, Cuevas-Ramos D, Fleseriu M. Medical management of Cushing's syndrome: current and emerging treatments. Drugs. 2019;79(9):935‐956. 31098899 10.1007/s40265-019-01128-7

[7] Braun LT, Reincke M. What is the role of medical therapy in adrenal-dependent Cushing's syndrome? Best Pract Res Clin Endocrinol Metab. 2020;34(3):101376. 32063487 10.1016/j.beem.2020.101376

[8] Fleseriu M, Castinetti F. Updates on the role of adrenal steroidogenesis inhibitors in Cushing's syndrome: a focus on novel therapies. Pituitary. 2016;19(6):643‐653. 27600150 10.1007/s11102-016-0742-1 PMC5080363

[9] Daniel E, Newell-Price JD. Therapy of endocrine disease: steroidogenesis enzyme inhibitors in Cushing's syndrome. Eur J Endocrinol. 2015;172(6):R263‐R280. 25637072 10.1530/EJE-14-1014

[10] Łebek-Szatańska A, Nowak KM, Zgliczyński W, Baum E, Żyłka A, Papierska L. Low-dose etomidate for the management of severe hypercortisolaemia in different clinical scenarios: a case series and review of the literature. Ther Adv Endocrinol Metab. 2019;10:2042018819825541. 30800267 10.1177/2042018819825541 PMC6378481

[11] Preda VA, Sen J, Karavitaki N, Grossman AB. Etomidate in the management of hypercortisolaemia in Cushing's syndrome: a review. Eur J Endocrinol. 2012;167(2):137‐143. 22577107 10.1530/EJE-12-0274

[12] Godefroi EF, Janssen PA, Van der Eycken CA, Van Heertum AH, Niemegeers CJ. DL-1-(1-arylalkyl)imidazole-5-carboxylate esters. A novel type of hypnotic agents. J Med Chem. 1965;8(2):220‐223. 14332665 10.1021/jm00326a017

[13] McGrath M, Raines DE. Anesthetic drug discovery and development: a case study of novel etomidate analogs. Methods Enzymol. 2018;603:153‐169. 29673523 10.1016/bs.mie.2018.01.026 PMC6945975

[14] Cherfan AJ, Arabi YM, Al-Dorzi HM, Kenny LP. Advantages and disadvantages of etomidate use for intubation of patients with sepsis. Pharmacotherapy. 2012;32(5):475‐482. 22488264 10.1002/j.1875-9114.2012.01027.x

[15] Ledingham IM, Watt I. Influence of sedation on mortality in critically ill multiple trauma patients. Lancet. 1983;321(8336):1270. 10.1016/s0140-6736(83)92712-5 6134053

[16] Preziosi P, Vacca M. Etomidate and corticotrophic axis. Arch Int Pharmacodyn Ther. 1982;256(2):308‐310. 7103618

[17] Fragen RJ, Shanks CA, Molteni A, Avram MJ. Effects of etomidate on hormonal responses to surgical stress. Anesthesiology. 1984;61(6):652‐656. 6095701 10.1097/00000542-198412000-00004

[18] Allolio B, Stuttmann R, Leonhard U, Fischer H, Winkelmann W. Adrenocortical suppression by a single induction dose of etomidate. Klin Wochenschr. 1984;62(21):1014‐1017. 6096626 10.1007/BF01711723

[19] Wagner RL, White PF, Kan PB, Rosenthal MH, Feldman D. Inhibition of adrenal steroidogenesis by the anesthetic etomidate. N Engl J Med. 1984;310(22):1415‐1421. 6325910 10.1056/NEJM198405313102202

[20] Hahner S, Stürmer A, Fassnacht M, et al Etomidate unmasks intraadrenal regulation of steroidogenesis and proliferation in adrenal cortical cell lines. Horm Metab Res. 2010;42(7):528‐534. 20352599 10.1055/s-0030-1249629

[21] Gärtner R, Albrecht M, Müller OA. Effect of etomidate on hypercortisolism due to ectopic ACTH production. Lancet. 1986;327(8475):275. 10.1016/s0140-6736(86)90811-1 2868286

[22] Forman SA, Warner DS. Clinical and molecular pharmacology of etomidate. J Am Soc Anesth. 2011;114(3):695‐707. 10.1097/ALN.0b013e3181ff72b5 PMC3108152 21263301

[23] Soh LM, Gunganah K, Akker SA, et al Etomidate in the emergency management of hypercortisolemia. Eur J Endocrinol. 2012;167(5):727‐728; author reply 729. 22930488 10.1530/EJE-12-0698

[24] Carlos R, Calvo R, Erill S. Plasma protein binding of etomidate in patients with renal failure or hepatic cirrhosis. Clin Pharmacokinet. 1979;4(2):144‐148. 455872 10.2165/00003088-197904020-00007

[25] Van de Wiele B, Rubinstein E, Peacock W, Martin N. Propylene glycol toxicity caused by prolonged infusion of etomidate. J Neurosurg Anesthesiol. 1995;7(4):259‐262. 8563446 10.1097/00008506-199510000-00004

[26] Doenicke A, Roizen MF, Nebauer AE, Kugler A, Hoernecke R, Beger-Hintzen H. A comparison of two formulations for etomidate, 2–hydroxypropyl-beta-cyclodextrin (HPCD) and propylene glycol. Anesth Analg. 1994;79(5):933‐939. 7978412 10.1213/00000539-199411000-00020

[27] Niedermirtl F, Eberhardt M, Namer B, et al Etomidate and propylene glycol activate nociceptive TRP ion channels. Mol Pain. 2018;14:1744806918811699. Doi: 10.1177/1744806918811699 30345869 PMC6856977

[28] https://figshare.com/articles/dataset/Muthukuda_et_al_JES_Supplementary_file_1_and_2_xlsx/28463927 [Supplementary file 1]

[29] https://figshare.com/articles/dataset/Muthukuda_et_al_JES_Supplementary_file_1_and_2_xlsx/28463927 [Supplementary file 2]

[30] Tasleem A, Cavaghan M, Czosnowski QA, Saeed Z. Use of continuous etomidate infusion to rapidly correct hypercortisolism in a patient with disseminated nocardiosis. Cureus. 2021;13(12):e20214. 35004034 10.7759/cureus.20214 PMC8729312

[31] Hays WB, Czosnowski Q. Continuous etomidate for the management of Cushing's syndrome complicated by pulmonary nocardiosis. J Pharm Pract. 2022;35(6):1057‐1059. 34056961 10.1177/08971900211017487

[32] Muthukuda D, Jayawickreme KP, Liyanarachchi K, et al Etomidate in the treatment of severe Cushing syndrome: the Sri Lankan experience. Sri Lanka J Diabetes Endocrinol Metab. 2024;15(2):5‐11.

[33] Moola SZ, Munn Z, Tufanaru C, et al Chapter 7: Systematic reviews of etiology and risk. In: Joanna Briggs Institute Reviewer's Manual. The Joanna Briggs Institute; 2019.

[34] Delavari S, Pourahmadi M, Barzkar F. What Quality Assessment Tool should I use? A practical guide for systematic reviews authors. Iran J Med Sci. 2023;48(3):229. 37791333 10.30476/IJMS.2023.98401.3038 PMC10542923

[35] Araujo Castro M, Palacios García N, Aller Pardo J, Izquierdo Alvarez C, Armengod Grao L, Estrada García J. Ectopic Cushing syndrome: report of 9 cases. Endocrinol Diabetes Nutr (Engl Ed). 2018;65(5):255‐264. 29655957 10.1016/j.endinu.2018.02.001

[36] Bucciarelli M, Lee YY, Magaji V. Cushing's storm secondary to a rare case of ectopic ACTH secreting metastatic breast cancer. Endocrinol Diabetes Metab Case Rep. 2015;2015(1):150051. 26525183 10.1530/EDM-15-0051 PMC4626655

[37] Tong CV, Hussein Z. Ectopic ACTH syndrome–experience with etomidate. J ASEAN Fed Endocr Soc. 2017;32(1):54‐56. 33442086 10.15605/jafes.032.01.10 PMC7784153

[38] Constantinescu SM, Driessens N, Lefebvre A, Furnica RM, Corvilain B, Maiter D. Etomidate infusion at low doses is an effective and safe treatment for severe Cushing's syndrome outside intensive care. Eur J Endocrinol. 2020;183(2):161‐167. 32449698 10.1530/EJE-20-0380

[39] Greening JE, Brain CE, Perry LA, et al Efficient short-term control of hypercortisolaemia by low-dose etomidate in severe paediatric Cushing's disease. Horm Res. 2005;64(3):140‐143. 16192738 10.1159/000088587

[40] Kalaria TR, Chopra R, Ayuk J, Buch H. Retinal vein occlusion as the presenting feature of Cushing's syndrome. BMJ Case Rep. 2021;14(1):e238204. 10.1136/bcr-2020-238204 PMC7839910 33495181

[41] Drake WM, Perry LA, Hinds CJ, Lowe DG, Reznek RH, Besser GM. Emergency and prolonged use of intravenous etomidate to control hypercortisolemia in a patient with Cushing's syndrome and peritonitis. J Clin Endocrinol Metab. 1998;83(10):3542‐3544. 9768661 10.1210/jcem.83.10.5156

[42] Carroll TB, Peppard WJ, Herrmann DJ, et al Continuous etomidate infusion for the management of severe Cushing syndrome: validation of a standard protocol. J Endocr Soc. 2019;3(1):1‐2. 30560224 10.1210/js.2018-00269 PMC6291660

[43] Krakoff J, Koch CA, Calis KA, Alexander RH, Nieman LK. Use of a parenteral propylene glycol-containing etomidate preparation for the long-term management of ectopic Cushing's syndrome. J Clin Endocrinol Metab. 2001;86(9):4104‐4108. 11549633 10.1210/jcem.86.9.7870

[44] Herrmann BL, Mitchell A, Saller B, et al [Transsphenoidal hypophysectomy of a patient with an ACTH-producing pituitary adenoma and an “empty sella” after pretreatment with etomidate]. Dtsch Med Wochenschr. 2001;126(9):232‐234. 11256037 10.1055/s-2001-11477

[45] Yun Castilla C, Rodríguez Amuedo F, Morales Martínez A, López Siguero JP, Martínez Aedo M, Milano Manso G. Usefulness of ethomidate in patients with Cushing syndrome with severe arterial hypertension and hypopotassemia. Med Intensiva. 2017;41(5):321‐322. 27461096 10.1016/j.medin.2016.05.005

[46] Kwon A, Choi Y, Jung JW, Suh J, Kim HS. Using etomidate in a two-month-old infant with Cushing syndrome due to adrenocortical carcinoma. J Clin Res Pediatr Endocrinol. 2022;14(1):102‐106. 33251784 10.4274/jcrpe.galenos.2020.2020.0164 PMC8900079

[47] Song K, Kwon A, Suh J, Choi HS, Chae HW, Kim HS. Cushing syndrome with acute kidney injury due to ureteral stones in a 6-year-old boy. Ann Pediatr Endocrinol Metab. 2020;25(4):277‐281. 32871646 10.6065/apem.2040026.013 PMC7788339

[48] Chan LF, Vaidya M, Westphal B, et al Use of intravenous etomidate to control acute psychosis induced by the hypercortisolaemia in severe paediatric Cushing's disease. Horm Res Paediatr. 2011;75(6):441‐446. 21430362 10.1159/000324419

[49] Mettauer N, Brierley J. A novel use of etomidate for intentional adrenal suppression to control severe hypercortisolemia in childhood. Pediatr Crit Care Med. 2009;10(3):e37‐e40. 19433940 10.1097/PCC.0b013e318198b096

[50] Baba M, Ray D. Severe psychosis due to Cushing's syndrome in a patient with a carcinoid tumour in the lung: a case report and review of the current management. World J Surg Oncol. 2015;13(1):165. 25926160 10.1186/s12957-015-0571-0 PMC4449963

[51] Bilgin YM, Van der Wiel HE, Fischer HR, De Herder WW. Treatment of severe psychosis due to ectopic Cushing's syndrome. J Endocrinol Invest. 2007;30(9):776‐779. 17993771 10.1007/BF03350817

[52] Hatta SFWM, Daly R, Chacko C, Raghavan R. A case of etomidate use in management of adrenocortical carcinoma with hypercortisolemia. J Endocrinol Metab. 2020;10(6):190‐194.

[53] Johnson TN, Canada TW. Etomidate use for Cushing's syndrome caused by an ectopic adrenocorticotropic hormone–producing tumor. Ann Pharmacother. 2007;41(2):350‐353. 17213295 10.1345/aph.1H365

[54] Cherian ET, Naik S, Cillo M, et al MON-902 etomidate-an under utilized but safe and efficacious drug to treat acute severe Cushing's Syndrome-case reports of ectopic ACTH syndrome from neuroendocrine malignancies. J Endocr Soc. 2020;4(Supplement_1):MON-902.

[55] Biller BM, Grossman AB, Stewart PM, et al Treatment of adrenocorticotropin-dependent Cushing's syndrome: a consensus statement. J Clin Endocrinol Metab. 2008;93(7):2454‐2462. 18413427 10.1210/jc.2007-2734 PMC3214276

[56] Jeffcoate WJ, Rees LH, Tomlin S, Jones AE, Edwards CR, Besser GM. Metyrapone in long-term management of Cushing's disease. Br Med J. 1977;2(6081):215‐217. 195666 10.1136/bmj.2.6081.215 PMC1631369

[57] Morgan FH, Laufgraben MJ. Mifepristone for management of Cushing's syndrome. Pharmacotherapy. 2013;33(3):319‐329. 23436494 10.1002/phar.1202

[58] Park SS, Kong SH, Yang YS, Ahn CH, Kim JH. Catatonia as a presenting symptom of ectopic adrenocorticotropic hormone syndrome caused by thymic carcinoid tumor. Pol Arch Intern Med. 2018;128(6):389‐391. 29968699 10.20452/pamw.4285

[59] Schulte HM, Benker G, Benker D, Sippell WG, Allolio B. Infusion of low dose etomidate: correction of hypercortisolemia in patients with Cushing's syndrome and dose-response relationship in normal subjects. J Clin Endocrinol Metab. 1990;70(5):1426‐1430. 2159485 10.1210/jcem-70-5-1426

[60] Allolio B, Schulte HM, Kaulen D, Reincke M, Jaursch-Hancke C, Winkelmann W. Nonhypnotic low-dose etomidate for rapid correction of hypercortisolaemia in Cushing's syndrome. Klin Wochenschr. 1988;66(8):361‐364. 3392892 10.1007/BF01735795

[61] Dabbagh A, Sa’adat N, Heidari Z. Etomidate infusion in the critical care setting for suppressing the acute phase of Cushing's syndrome. Anesth Analg. 2009;108(1):238‐239. 19095856 10.1213/ane.0b013e318187ed37

[62] Wong SWP, Yap YW, Narayanan RP, et al Etomidate in the management of severe Cushing's disease and MRSA bacteraemia in a district general hospital in the United Kingdom. Endocrinol Diabetes Metab Case Rep. 2019;2019(1):EDM190044. 31967974 10.1530/EDM-19-0044

[63] Yan JY, Nie XL, Tao QM, Zhan SY, De Zhang Y. Ketoconazole associated hepatotoxicity: a systematic review and meta-analysis. Biomed Environ Sci. 2013;26(7):605‐610. 23895707 10.3967/0895-3988.2013.07.013

